# Identification and Characterisation of the Antimicrobial Peptide, Phylloseptin-PT, from the Skin Secretion of *Phyllomedusa tarsius*, and Comparison of Activity with Designed, Cationicity-Enhanced Analogues and Diastereomers

**DOI:** 10.3390/molecules21121667

**Published:** 2016-12-03

**Authors:** Yitian Gao, Di Wu, Xinping Xi, Yue Wu, Chengbang Ma, Mei Zhou, Lei Wang, Mu Yang, Tianbao Chen, Chris Shaw

**Affiliations:** 1 Natural Drug Discovery Group, School of Pharmacy, Queen’s University, Belfast BT9 7BL, Northern Ireland, UK; ygao07@qub.ac.uk (Y.G.); dwu03@qub.ac.uk (D.W.); x.xi@qub.ac.uk (X.X.); ywu16@qub.ac.uk (Y.W.); c.ma@qub.ac.uk (C.M.); m.zhou@qub.ac.uk (M.Z.); t.chen@qub.ac.uk (T.C.); chris.shaw@qub.ac.uk (C.S.); 2 Liaoning Center for Certification of Drug, No.7 Chongshanxi Road, Huanggu District, Shenyang 1110036, China

**Keywords:** antimicrobial peptide, phylloseptin, stability, diastereomer, modification

## Abstract

Antimicrobial peptides belonging to the phylloseptin family are mainly found in phyllomedusine frogs. These peptides not only possess potent antimicrobial activity but exhibit low toxicity against eukaryotic cells. Therefore, they are considered as promising drug candidates for a number of diseases. In a recent study, potent antimicrobial activity was correlated with the conserved structures and cationic amphiphilic characteristics of members of this peptide family. A phylloseptin peptide precursor was discovered here in the skin secretion of *Phyllomedusa tarsius* and the mature peptide was validated by MS/MS sequencing, and was subsequently named phylloseptin-PT. The chemically-synthesized and purified phylloseptin-PT displayed activity against *Staphylococcus aureus* and *Candida albicans*. Nevertheless, a range of cationicity-enhanced peptide analogues of phylloseptin-PT, which contained amino acid substitutions at specific sites, exhibited significant increases in antimicrobial activity compared to native phylloseptin-PT. In addition, alternative conformers which were designed and chemically-synthesized with d-lysine, showed potent antimicrobial activity and enhanced bioavailability. These data indicate that phylloseptins may represent potential candidates for next-generation antibiotics. Thus, rational design through modification of natural antimicrobial peptide templates could provide an accelerated path to overcoming obstacles en-route to their possible clinical applications.

## 1. Introduction

The drug market, in recent centuries, has been dominated by small molecules because of their specific desirable characteristics such as potent pharmacological activity, deterministic pharmacodynamics, and rapid analysis [1]. However, this situation is steadily changing. The expanding worldwide pharmaceutical market has been increasingly investing in peptides/proteins over the past few years because such drugs have made significant gains in both diagnostics and therapeutics due to technological breakthroughs and advances [2]. Among the different categories of drug candidates, antimicrobial peptides are considered as the best alternatives to conventional antibiotics for use against resistant microbes with high therapeutic indices [3,4,5]. It has been speculated that more than 2000 host defence peptides have been discovered and characterized and most of these have been demonstrated to exert prominent bacteriostatic and bactericidal effects [6]. As the most promising antibiotic substitutes, there are still several obstacles and drawbacks to peptide drugs which are limiting their clinical applications and which urgently need to be addressed and solved. Peptide drugs are prone to degradation by gastrointestinal proteases through oral delivery and they are susceptible to enzymatic degradation even after they enter systemic circulation [7]. This problem can seriously impact their therapeutic index. Therefore, the task of combining the potent antimicrobial activity of a peptide with improved stability and bioavailability has become the goal of future research on these molecules. 

Phylloseptins are a family of potent antimicrobial peptides that are widely found in the skin secretions of phyllomedusine frogs [8]. The structures of these peptides are relatively conserved, containing 19–21 amino acids with C-terminal amidation, a cationic amphiphilic structure and an α-helical domain [9]. Key to their potent antimicrobial activity is this conserved structure, which is thought to disrupt the cytoplasmic membrane rather than combine with specific targets or receptors [10]. Indisputably, antimicrobial peptides have short half-lives, low bioavailability, and high costs but these drawbacks can be overcome by sustained efforts in the discovery, development, and rational modification of the peptides [7,11,12,13]. 

This study describes the isolation of a novel antimicrobial peptide precursor from the *Phyllomedusa tarsius* skin secretion using a high-throughput method of “shotgun” molecular cloning combined with mass spectrometry. Several cationicity-enhanced peptide analogues were designed, which preserved conserved sequences while enhancing positive charges. Here, one potent antimicrobial peptide analogue was chosen as a template and two synthetic diastereomers, composed of d-amino acid residue substitutions in specific positions, were used to investigate the effect of enhancing the stability of the native peptide. 

## 2. Results

### 2.1. Molecular Cloning and Identification of Phylloseptin-PT Precursor-Encoding cDNA from the Skin Secretion of P. tarsius 

**Figure 1:**
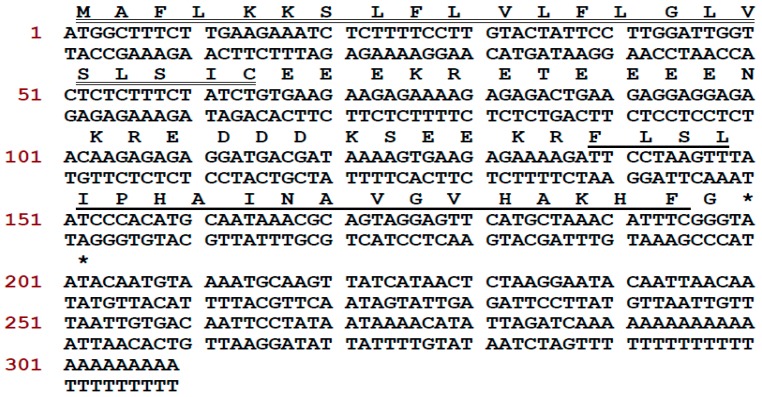
Nucleotide and translated open-reading frame amino acid sequences of cloned cDNA encoding the precursor of Phylloseptin-PT (PS-PT). The putative signal peptide is double-underlined, the mature peptide is single-underlined, and the stop codon is indicated by an asterisk.

A novel antimicrobial peptide precursor-encoding cDNA was cloned from the *Phyllomedusa tarsius* skin secretion and the translated peptide precursor was identified as encoding a member of the phylloseptin family. The full-length cDNA was cloned from the skin secretion-derived cDNA library of *Phyllomedusa tarsius*, employing a degenerate primer (S1; 5′-ACTTTCYGAWTTRYAAGMCCAAABATG-3′) (Y = C + T, W = A + T, R = A + G, M = A + C, B = T + C + G), which was designed according to the regions of highly-conserved peptide transcripts from related phyllomedusine frog species, and a universal primer. The nucleic acid and translated amino acid sequence of the novel phylloseptin-encoding precursor contained an open-reading frame of 66 amino acid residues (Figure 1). The translated open-reading frame consisted of a putative signal peptide (MAFLKKSLFLVLFLGLVSLSIC) of 22 amino acid residues, a 22-residue acidic “spacer” domain, and a 19-residue phylloseptin-encoding domain. This peptide precursor shows a high degree of similarity to a previously discovered member of the phylloseptin family, named phylloseptin-PT (PS-PT). In accordance with other phylloseptins, phylloseptin-PT is cleaved from its precursor by propeptide convertase cleavage at classical sites consisting of paired Lys-Arg (K-R) amino acid residues at its N-terminus. The C-terminal glycine residue is post-translationally modified as an amide donor which is a common feature in the phylloseptin family of peptides and the resultant amide is considered to be essential for antimicrobial activity [10]. The nucleotide sequence of this phylloseptin-PT precursor has been registered in the European Molecular Biology Laboratory (EMBL) Nucleotide Sequence Database under the accession code, LT591888. This mature peptide sequence was identified previously by using mass spectrometry and named phylloseptin-13, but the full precursor was first discovered from the skin secretion of *Phyllomedusa tarsius* and confirmed the structural characterization [10].

**Figure 2:**
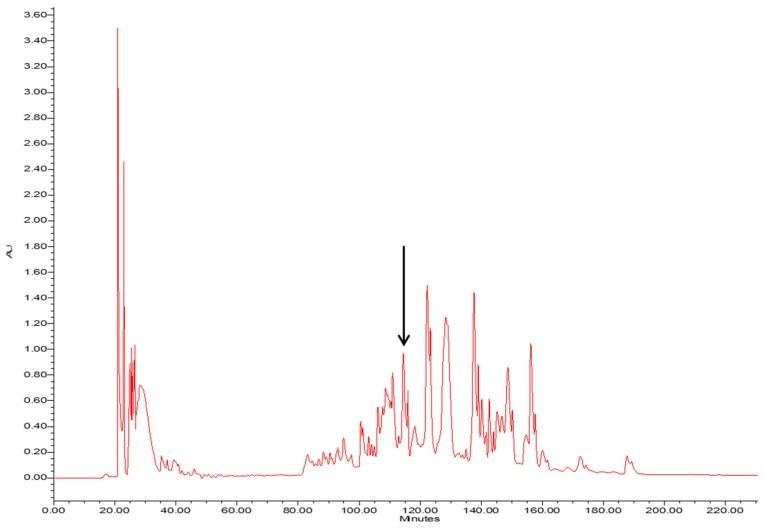
The RP-HPLC chromatogram of the skin secretion of *Phyllomedusa tarsius*. The components were monitored at a wavelength of 214 nm. The retention time of phylloseptin-PT is marked by an arrow.

**Figure 3:**
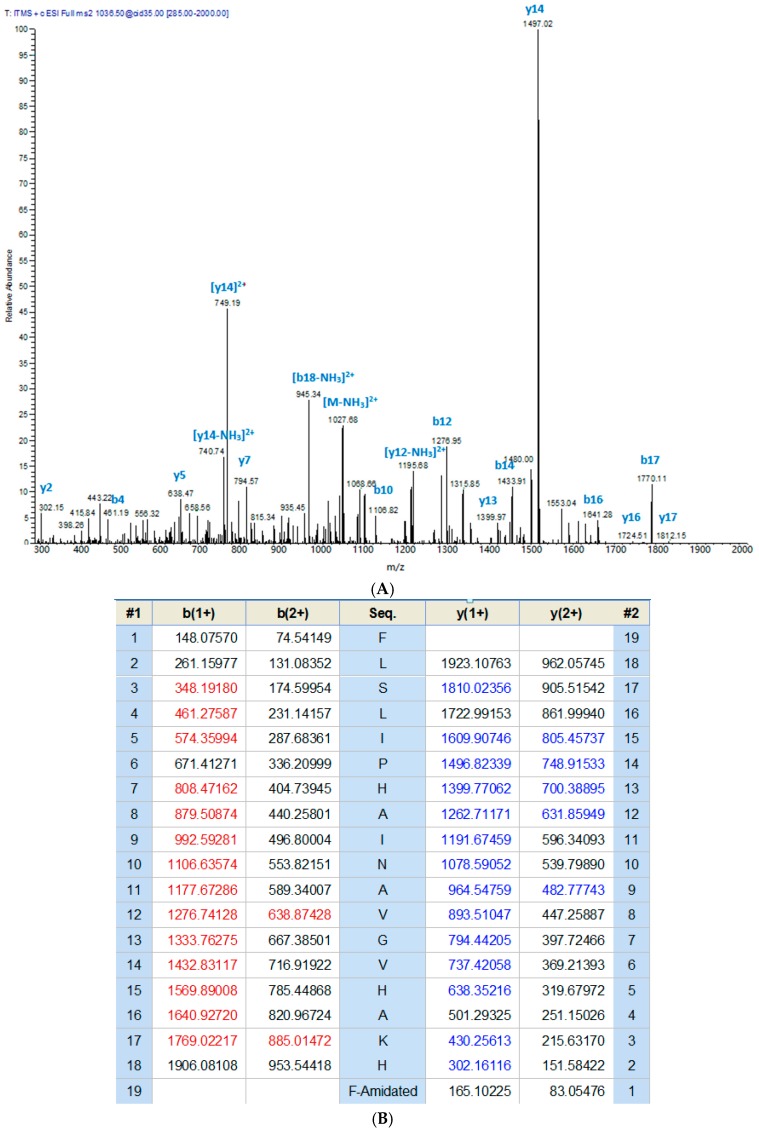
Ion spectrum of phylloseptin-PT (**A**) MS/MS data from collision-induced dissociation of the doubly-charged precursor ion (1036.50 *m*/*z*). (**B**) The calculated fragment ions from the sequence with those observed following MS/MS indicated in blue and red typefaces.

The crude skin secretion of *Phyllomedusa tarsius* was analysed by a combination of reverse-phase high performance liquid chromatography (RP-HPLC) and matrix-assisted laser desorption/ionisation-time of flight mass spectrometer (MALDI-TOF/MS). The elution position/retention time of a peptide with a mass corresponding to that predicted for phylloseptin-PT, is indicated in the chromatogram shown in Figure 2. The phylloseptin-PT, whose molecular mass was identical to that of the putative antimicrobial peptide from cloned cDNA, was subjected to primary structural validation by use of MS/MS fragmentation. The fragment ions (Figure 3), which were obtained by ion trapping in the electrospray mass spectrometer, were analysed by aligning with entries in the database. In addition, the modification of C-terminal amidation was found in the mass spectrometric analysis, which was consistent with the presence of a glycine amidation motif in the propeptide as determined through molecular cloning. This peptide and other designed analogue/diastereomer peptides were chemically synthesized and purified by reverse-HPLC to obtain sufficient quantities for functional bioassays.

### 2.2. Design, Synthesis, Predicted Physicochemical Parameters, and Secondary Structures of Peptides

**Figure 4:**
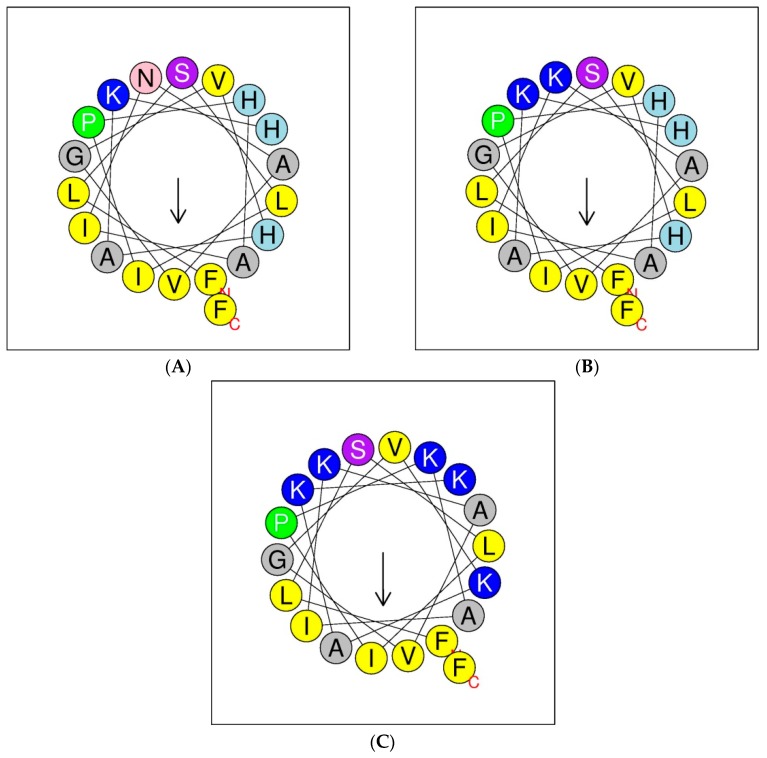
Putative secondary structures of PS-PT (**A**), PS-PT1 (**B**) and PS-PT2 (**C**) described by helical wheel projections. Arrows denote the direction of the hydrophobic moments.

**Figure 5:**
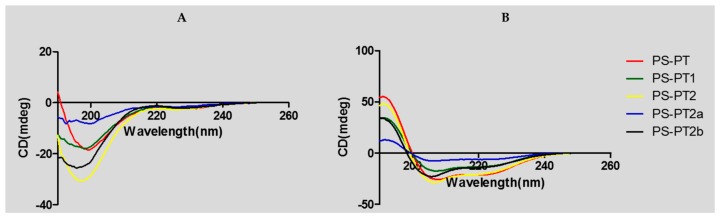
Circular dichroism (CD) spectra of PS-PT and its analogues (100 μM) (**A**) in 10 mM ammonium acetate water solution and (**B**) in 50% 2,2,2-trifluoroethanol (TFE)/10 mM ammonium acetate water solution.

Helical wheel projections of the phylloseptin and its designed analogues are shown below in Figure 4. The predicted secondary structures and physiochemical parameters of the peptides are summarised in Table 1. Five phylloseptin peptides were chemically synthesized, purified by RP-HPLC, and structurally validated by mass spectrometry. Noticeably, phylloseptin-PT2a (PS-PT2a) and phylloseptin-PT2b (PS-PT2b) are two conformers of phylloseptin-PT (PS-PT2). All the peptides contained amphipathic structures and an overall positive charge. The structural design enhanced the cationic charges. The secondary structure of each peptide was determined in circular dichroism (CD). All the peptides showed random coil structures in 10 mM ammonium acetate solution (Figure 5A), while they exhibited typical α-helical structures in the membrane mimetic environment (Figure 5B). The helicity was calculated by the K2D3 web server and summarized in Table 1.

### 2.3. Antimicrobial and Haemolysis Assays

**Figure 6:**
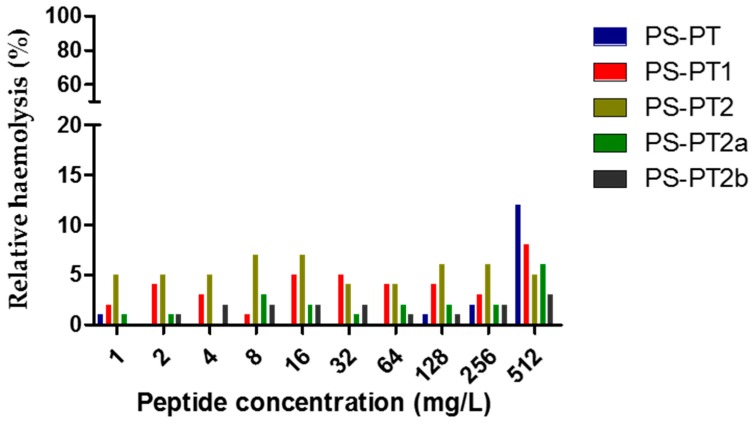
Relative haemolysis of PS-PT and its analogues. The 100% haemolysis was induced by 1% Triton X-100.

The antimicrobial activities of all the peptides were assessed and the results were summerised in Table 2. Generally, the natural peptide, phylloseptin-PT, was inhibitory against two selected organisms, *S. aureus* and *C. albicans*, while the cationicity-enhanced analogues, PS-PT1 and PS-PT2, displayed an enhanced antimicrobial activity of broader spectrum. PS-PT1 showed a 4-fold enhanced inhibition against the three selected organisms. PS-PT2 exhibited dramatically enhanced inhibitory activity against reference organisms, increasing the inhibition by 32-fold, with a range of 8–16 mg/L against *C. albicans*. However, PS-PT2a, the d-lysine substituted analogue of PS-PT2, nearly lost all antimicrobial activity against reference organisms, only sustaining inhibitory activity against *C. albicans*. Differing from PS-PT2a, PS-PT2b maintained potent antimicrobial activity, especially showing intense inhibition against Gram-negative *E. coli*. Nonetheless, no haemolytic effect was detected for all five phylloseptin peptides (Figure 6). Thus, some designed peptides could enhance antimicrobial activities without increasing haemolysis, consequently improving the therapeutic index.

### 2.4. Comparison of the Stability of Diastereomers

The degradation of wild-type PS-PT2 was rapid, both by horse serum and trypsin (Table 3). The original peptide was completely cleaved by trypsin in 30 s and the Lys^7^-Ala^8^ scissile bond was the most susceptible to trypsin cleavage. For PS-PT2, some other fragments of 1–17 were detected by incubating with trypsin for 4 min. However, for PS-PT2a, as all the L-lysines in the sequence were substituted by d-isomers, it was stable in trypsin solution for up to 48 h. In the serum environment, PS-PT2 was completely degraded after 30 min. The catabolite oligopeptides of PS-PT2 were mainly the 8–19 fragment (-AIKAVGVKAKKF) by cleavage at the Lys^7^-Ala^8^ position and the process of this degradation was sustained for up to 36 h. A small quantity of the 1–16 fragment (FLSLIPKAIKAVGVKA-) was seen after 1 min and persisted to 30 min. Another small quantity of the 3–19 fragment (-LSLIPKAIKAVGVKAKKF-) simultaneously appeared during this period. However, PS-PT2a was relatively stable after 72 h. Compared with the wild-type peptide, only a small amount of the 3–19 fragment (LSLIPKAIKAVGVKAKKF) was observed after 4 min as the main degradation pathway, indicating that endopeptidase was the predominant protease. Small quantities of 2–7 fragments (LSLIPK) were subsequently found after a 4 h incubation and kept increasing until the experiment was terminated. For PS-PT2b, although the l-lysine in the seventh position was substituted by d-lysine, the peptide analogue was still hydrolytically-degraded after a 30 s incubation with trypsin. However, the scissile site was changed to Lys^10^-Ala^11^ compared to the original site Lys^7^-Ala^8^. This is because trypsin specifically targets l-conformed positively-charge amino acids. Thus, even though the 7-lysine was substituted with a d-enantiomer, other l-conformed positive sites can be cleaved. However, PS-PT2b exhibited strong stability in a serum environment compared with the wild-type peptide PS-PT2. In addition, the fragments, 1–15 (FLSLIPKAIKAVGVK), 1–14 (FLSLIPKAIKAVGV), and 1–17 (FLSLIPKAIKAVGVKAK), were observed only after prolonged incubation (>48 h). 

## 3. Discussion

**Figure 7:**
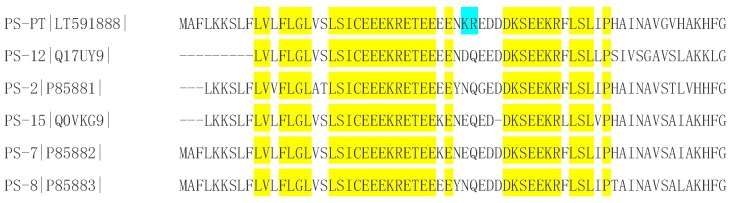
Alignment of precursors of PS-PT and other phylloseptin peptides isolated from *Phyllomedusa hypochondrialis azurea*. Consensus residues are highlighted in yellow and specific sign is indicated in blue.

Following cloning and DNA sequencing, a novel peptide precursor was cloned from the defensive skin secretion of *Phyllomedusa tarsius*. The mature peptide sequence was found to end with a glycine residue, serving as an amide donor in the process of post-translational modification. As the observed mass-to-charge (*m*/*z*) ratio of the y2-ion was found to be 1 Da less than calculated, this confirmed the presence of the amide modification at the C-terminus. Interestingly, this mature peptide has been previously reported as PS-13 from the skin secretion of *Phyllomedusa hypochondrialis azurea*, and has been successfully identified by mass spectrometry without the cDNA encoding sequence [10]. Therefore, the comparison of the precursors between PS-13 and PS-PT was not able to be accomplished. Considering that other phylloseptin precursors are highly conserved, we compared the PS-PT precursor with other phylloseptin peptide precursors from *Phyllomedusa hypochondrialis azurea*. The precursor of PS-PT is similar to the others except for a typical basic dipeptide, -KR-, in the middle (positions 35 and 36) of the putative acidic “spacer” region, instead of acidic and amidic residues (Figure 7). It could be a sign to differentiate the phylloseptin precursors from *Phyllomedusa hypochondrialis azurea* and *Phyllomedusa tarsius*. Meanwhile, in this study, molecular cloning was offered to validate the accurate sequences, as mass spectrometry might be of low confidence by the differentiation of leucine and isoleucine. So we considered illustrating the higher confidence of identification as well as the novelty of its precursor. This is the first phylloseptin identified from *Phyllomedusa tarsius*. PS-PT showed low antimicrobial activity against *S. aureus* and *C. albicans* at 512 mg/L and no effect against the growth of *E. coli*. Consistently, it exhibited weakly haemolytic activity on horse erythrocytes**.** To increase the potency of antimicrobial activity and overcome some drawbacks such as toxicity to eukaryotic cells and a short half-life in vivo, the analogues of PS-PT were designed and subsequently investigated. 

In the phylloseptin family, most peptides exhibit a potent antimicrobial activity due to their relatively conserved primary structures. Most contain 19–20 amino acids, several of which are histidine and/or lysine, a factor which favours attachment to cell membranes by electrostatic forces [14]. A highly conserved motif, Phe-Leu-Ser-Leu-Ile/Leu-Pro-, at the N-terminus, relates to this function [15]. The proline at position 6 is important because it is considered to distort the linear structure [16] by introducing a slight bend in the backbone of the structure to avoid steric effects of the side chains of backbone residues, although simultaneously retaining the amphipathic character [10]. Although this segment breaks the alpha-helix, nevertheless it has functional relevance as it may enhance activity and selectivity [17]. The amidated C-terminal region is more hydrophilic and cationic and both of these characteristics could also contribute to improving antimicrobial activity [18]. The distribution of hydrophobic and hydrophilic amino acids present on the helical wheel showed the essential amphipathic character of PS-PT (Figure 4). However, the predicted parameters revealed that the amphipathic α-helical region of PS-PT, which was considered as the predominant region disturbing membrane permeability, was quite low and this could result in low antimicrobial activity. Considering all the above and some other common impacting factors such as amphipathicity and net positive charges, the first modified peptide contained a substitution of Asn^10^ to an electropositive Lys, and was named PS-PT1. Antimicrobial activities against the selected test microorganisms were correspondingly increased by 4-fold over the natural peptide PS-PT. These results confirmed the hypothesis that these cationic peptides interact with anionic phospholipids of the bacterial cell membrane through electrostatic forces [19].

Another cationicity-enhanced analogue was modified by substituting His residues (at positions 7, 15, and 18) and Asp (at position 10) with Lys, and was named PS-PT2. This design combined the conserved motif with enhanced cationicity. Additionally, the hydrophobic moment of PS-PT2, an important structural characteristic for α-helical antimicrobial peptides, was higher than the other peptide analogues. This design is suggested to stabilise the amphipathic structure and ensures a proper residue distribution in the hydrophobic and hydrophilic faces in the helical wheel. The antimicrobial activity of this designed analogue was significantly increased by 32-fold against *C. albicans* and by 8-fold against *S. aureus,* in addition to displaying negligible haemolytic effects.

Nonetheless, the stability of natural peptides is a major reason for developing novel drug delivery methods, as peptides are easily degraded into small fragments or amino acids by various endogenous enzymes. However, it has been reported that the replacement of l-lysine by d-lysine may maintain potent antimicrobial activity and provide resistance to protease degradation [20]. In this study, d-lysine was used to synthesize an optical isomer using PS-PT2 as a template and this peptide was named PS-PT2a. Incubation of PS-PT2 and PS-PT2a with horse serum and trypsin, respectively, revealed that PS-PT2 was degraded rapidly at the most susceptible site of l-lysine at position 7 in a few minutes, while the d-lysine substituted peptide was barely degraded at all over 48 h. This result indicates that the antimicrobial potency of PS-PT2 might be gone within a few minutes in vivo and this obviously limits its effects, while the antimicrobial activity of those fragments could be further investigated. The antimicrobial assay showed that the antimicrobial activity of PS-PT2a is defective compared to PS-PT2. It could be explained by the fact that the d-amino acids affect the secondary structure, especially the α-helix in this sequence. This has clearly shown that the α-helicity of PS-PT2a declined dramatically compared to PS-PT2, from 37.91% to 1.06%. Meanwhile, it has been proposed that increasing net positive charge of a peptide in a neutral environment is a good approach to enhance antimicrobial activity, but the maintenance of amphipathicity and the helical domain are more significant.

An alternative method to overcome this limitation is to replace the amino acid in the most protease susceptible site with a d-amino acid. In this study, the l-lysine at position 7 was replaced by its d-isomer and the peptide was named PS-PT2b. This peptide retained its antimicrobial activity and stability in horse serum. The retained activity of PS-PT2b was less potent than PS-PT2, especially against the Gram positive bacterium, *S. aureus*. It was presumed that Lys-7 had a high propensity for forming an α-helix and that this α-helical structure was required for activity against Gram positive bacteria. The result of the CD has confirmed this, assuming that the α-helicity of PS-PT2b was slightly less than PS-PT2, about 23.92%. The α-helicity sustaining in a certain range is a necessary condition for the maintenance of potent antimicrobial activity. These data offered an insight into a possible strategy for the design of antimicrobial peptides against selected bacteria by the use of d-amino acid substitutions.

## 4. Materials and Methods 

### 4.1. Acquisition of Phyllomedusa Tarsius Skin Secretions

The *Phyllomedusa tarsius* frogs were acquired from a commercial source (PeruBiotech E.I.R.L., Lima, Peru) and the skin secretions were obtained by mild transdermal electrical stimulation [21]. Following stimulation, skin secretions were collected by rinsing with deionized water and were then snap frozen in liquid nitrogen, freeze-dried, and stored at −20°C for future study.

### 4.2. Molecular Cloning of Phylloseptin-PT Precursor-Encoding cDNA from a Skin Secretion-Derived cDNA Library of P. tarsius

The isolation of pure mRNA from crude skin secretion was achieved by utilizing a magnetic oligo-dT bead kit (Dynal Biotech, Merseyside, UK) which could bind polyadenylated mRNA in the cell lysis buffer supplied with the kit. Reverse transcription and synthesis of first-strand cDNA was followed by a 3’-RACE reaction to isolate target antimicrobial peptide precursor nucleic acid sequence data with a SMART-RACE kit (Clontech, Palo Alto, CA, USA). 3’-RACE was facilitated by a nested universal primer (NUP) (supplied by the kit) and a sense primer (S1; 5′-ACTTTCYGAWTTRYAAGMCCAAABATG-3′) (Y = C + T, W = A + T, R = A + G, M = A + C, B = T + C + G) which was designed according to the highly conserved segment of the 5′-untranslated region of phylloxin cDNA from *Phyllomedusa bicolor* and the opioid peptide cDNA from *Phyllomedusa dacnicolor* [22,23]. The PCR cycling procedure included an initial denaturation step at 94 °C maintained for 90 s, then 35 thermal cycles which involved 60 s at 94 °C for denaturation, primer annealing for 30 s at 58 °C, and 180 s for extension at 72 °C. The PCR products were purified by gel electrophoresis and cloned using a pGEM-T vector system (Promega Corporation, Southampton, UK). The DNA sequences of clones were obtained by use of an ABI 3100 automated capillary sequencer (Applied Biosystems, Foster City, CA, USA).

### 4.3. Identification and Structural Characterization of the Putative Antimicrobial Peptide

Five mg of lyophilised skin secretion were dissolved, clarified, and injected into an HPLC system (Waters, Milford, MA, USA) to separate the fractions using a gradient programme which ran over 240 min at a flow rate of 1 mL/min from water/TFA (99.95/0.05, *v*/*v*) to acetonitrile/water/TFA (80/19.95/0.05; *v*/*v*/*v*) on an analytical column (Jupiter C5, 5 μm, 240 mm × 4.6 mm, Phenomenex, Macclesfield, Cheshire, UK). The effluent was constantly monitored by a UV detector set at 214 nm (λ) and the fractions were automatically collected at minute intervals. All fractions were analysed by MALDI-TOF/MS (Voyager DE, Perspective Biosystems, Foster City, CA, USA) with CHCA as the matrix in positive mode. The instrument was calibrated by standards and set accuracy was ±0.1%. The peptide with a molecular mass coincident with that predicted from cloned cDNA, was injected into an LCQ-Fleet electrospray ion-trap mass spectrometer to analyse its primary structure by MS/MS fragmentation (Thermo Fisher Scientific, San Francisco, CA, USA).

### 4.4. Determination of Peptide Secondary Structures and Prediction of Antimicrobial and Physicochemical Properties

The secondary structure of each peptide was determined using a JASCO J-815 CD spectrometer (Jasco, Essex, UK). Each peptide was dissolved in 10 mM ammonium acetate and 10 mM ammonium acetate with 50% TFE, respectively and was prepared at 100 μM in a 1 mm high precision quartz cell (Hellma Analytics, Essex, UK). CD spectra were recorded at a wavelength ranging from 190 nm to 250 nm with a 100 nm/min scan speed. The parameters were set as 1 nm bandwidth and 0.5 nm data pitch. The result was analysed by the K2D3 webserver to estimate α-helical content [24]. The helical wheel projections and significant physiochemical parameters of peptides, were predicted by use of Network Protein Sequence Analysis [25] and Heliquest [26] programmes. The helical wheel projection was utilized to describe the properties of alpha helices as a visual plot [27]. The significant parameters such as hydrophobicity, hydrophobic moment, and net charge at neutral pH, which were considered as significant factors correlating with antimicrobial activity, antimicrobial spectrum, and haemolysis, were also predicted and calculated [28,29,30,31,32].

### 4.5. Peptide Synthesiss

The natural peptide and respective cationicity-enhanced analogues, were chemically-synthesized by an automatic solid-phase synthesiser (Protein Technologies, Tucson, AZ, USA) with natural l-amino acids, while d-lysine substituted cationicity-improved peptides were synthesized with d-lysine and other l-amino acids in their sequences. The process was carried out by standard Fmoc chemical synthesis, deprotection, and cleavage procedures. Each peptide was purified by HPLC and structurally-validated by mass spectrometry.

### 4.6. Antimicrobial Assay

The antimicrobial activity was evaluated by minimal inhibitory concentration (MIC) and minimum bactericidal concentration (MBC) assays using broth microdilution methods and in vitro assays against reference strains of microorganisms; a Gram-positive bacterium, a Gram-negative bacterium, and a yeast, respectively, *Staphylococcus aureus* (NCTC 10788), *Escherichia coli* (NCTC 10418), and *Candida albicans* (NCPF 1467). Briefly, the reference strains were incubated in Muller-Hinton Broth (MHB) until logarithmic phase growth was achieved which was assessed by the optical density (OD) of the culture at 550 nm. The cultures were subjected to dilution to obtain 1 × 10^6^ colony-forming units (cfu)/mL for bacteria or to 5 × 10^5^ cfu/ml for the yeast. Serial peptide dilutions and cultures were loaded into a 96-well microtiter plate obtaining suspensions containing 1–512 mg/L tested peptides. After incubating at 37 °C for 24 h, MIC values were determined as the lowest concentration which produced a similar OD value to the negative control (medium only). Then 10 μL of inhibited culture from each well was subcultured on Muller-Hinton Agar (MHA) and incubated for 24 h. The MBC values were defined as the minimum concentration which effected no growth.

### 4.7. Haemolysis Assay

The haemolytic activity of peptides against defibrinated horse erythrocytes (TCS Biosciences Ltd., Botolph Claydon, Buckingham, UK) was determined by comparing the degree of lysis after incubating tested peptides with prewashed red cell suspensions for 2 h. A 2% suspension (*v*/*v*) was formed with prewashed defibrinated horse erythrocytes and sodium phosphate-buffered saline (PBS). Peptides were incubated with this 2% suspension in a final concentration range from 1 to 512 mg/L and all the samples were kept at a constant 37 °C for 120 min. Negative controls and positive controls were PBS alone and PBS containing Triton X-100 (Sigma-Aldrich, St. Louis, MO, USA), respectively. The sample supernatants were used to assess the extent of haemolysis by measuring the OD value at 550 nm.

### 4.8. Stability and Degradation of Peptides

Two different media were used to appraise the stability and degradation sites of peptides, trypsin, and horse serum, respectively. For the trypsin assay, 1 mg of trypsin (Sigma-Aldrich) with 1 mg of peptide were incubated in 1 mL PBS for 72 h. Whilst for horse serum, the medium was prepared with 25% horse serum/RPMI medium (*v*/*v*) and incubated at 37 °C for 15 min. Peptide stock solution was added into the serum medium to obtain a solution having a final concentration of 1 mg/mL and test samples were kept at 37 °C for 72 h. At different time points, 20 μL samples were transferred into 100 μL of 0.25% (*v*/*v*) TFA/water which caused reaction termination. For the serum-digestion, samples were kept at 4 °C and centrifuged at 13,000 rpm for 15 min and the supernatants were then analysed by MALDI-TOF/MS.

## 5. Conclusions

This work describes the isolation and structural elucidation of an antimicrobial peptide from the *Phyllomedusa tarsius* skin secretion, utilizing a high throughput system combining mass spectrometric sequencing and “shotgun” molecular cloning to obtain the cDNA sequence. Following this, the study progressed by assessing the bioactivity, toxicity, and stability of the peptide. Four cationicity-enhanced peptide analogues were designed, chemically synthesized, and evaluated. The cationicity-enhanced peptides exhibited more potent antimicrobial activity compared to the natural peptide. The data demonstrated that site-selective amino acid substitutions with d-isomers could sustain a similar antimicrobial activity with prolonged stability. Generally, these studies elucidated a method to discover and evaluate novel natural antimicrobial peptides, and to design and synthesise analogues with enhanced potency, specific activity, and stability to potentially develop as novel therapeutic agents.

## Figures and Tables

**Table 1:** The amino acid sequences of PS-PT and analogues with their physicochemical parameters. The predicted secondary structures of PS-PT, PS-PT1, and PS-PT2 are shown below each peptide sequence, respectively. d-type amino acids are indicated in bold typeface.

Peptide	Sequence	Hydrophobicity (H)	Hydrophobic Moment (μH)	% Helix	Net Charge
PS-PT	FLSLIPHAINAVGVHAKHF-NH_2_	0.707	0.418	37.92	2
PS-PT1	FLSLIPHAIKAVGVHAKHF-NH_2_	0.686	0.438	19.48	3
PS-PT2	FLSLIPKAIKAVGVKAKKF-NH_2_	0.509	0.497	37.91	6
PS-PT2a	FLSLIP**K**AI**K**AVGV**K**A**KK**F-NH_2_	0.509	0.497	1.06	6
PS-PT2b	FLSLIP**K**AIKAVGVKAKKF-NH_2_	0.509	0.497	23.92	6

**Table 2:** Minimum inhibitory concentrations (MICs) and minimum bactericidal concentrations (MBCs) of PS-PT and its derivatives as determined for specified microorganisms.

Peptide	MIC (mg/L)			MBC (mg/L)		
	*S. aureus*	*E. coli*	*C. albicans*	*S. aureus*	*E. coli*	*C. albicans*
PS-PT	512	>512	512	>512	>512	>512
PS-PT1	128	512	128	256	>512	256
PS-PT2	64	512	16	64	>512	32
PS-PT2a	>512	>512	256	>512	>512	256
PS-PT2b	256	256	32	512	256	64

**Table 3:** Catabolites generated by incubation of PS-PT2 and its d-type analogues with trypsin or serum.

Conditions	PS-PT2	PS-PT2a	PS-PT2b
Trypsin	1–19 FLSLIPKAIKAVGVKAKKF	1–19 FLSLIP**K**AI**K**AVGV**K**A**KK**F	1–19 FLSLIP**K**AIKAVGVKAKKF
	1–7 FLSLIPK		1–10 FLSLIP**K**AIK
	8–19 AIKAVGVKAKKF		11–19 AVGVKAKKF
	1–17 FLSLIPKAIKAVGVKAK		
Serum	1–19 FLSLIPKAIKAVGVKAKKF	1–19 FLSLIP**K**AI**K**AVGV**K**A**KK**F	1–19 FLSLIP**K**AIKAVGVKAKKF
	1–7 FLSLIPK	3–19 SLIP**K**AI**K**AVGV**K**A**KK**F	1–14 FLSLIP**K**AIKAVGVK
	8–19 AIKAVGVKAKKF	2–7 LSLIP**K**	1–15 FLSLIP**K**AIKAVGVKA
	1–16 FLSLIPKAIKAVGVKA		1–17 FLSLIP**K**AIKAVGVKAK
	3–19 SLIPKAIKAVGVKAKKF		

## Abbreviations

The following abbreviations were used in this manuscript:

RP-HPLC:	Reverse-phase high-performance liquid chromatography
MALDI-TOF/MS:	Matrix-assisted laser desorption/ionization time-of-flight mass spectrometry
CHCA:	α-cyano-4-hydroxycinnamic acid
RACE:	Rapid Amplification of cDNA Ends
Fmoc:	9-fluorenylmethyloxycarbonyl
TFA:	Trifluoroacetic Acid
